# Bis(2-amino-3-methyl­pyridine)­dichlorido­cobalt(II)

**DOI:** 10.1107/S1600536810036597

**Published:** 2010-09-25

**Authors:** Azadeh Tadjarodi, Keyvan Bijanzad, Behrouz Notash

**Affiliations:** a Department of Chemistry, Iran University of Science and Technology, Tehran 16846-13114, Iran; b Department of Chemistry, Shahid Beheshti University, G. C., Evin, Tehran 1983963113, Iran

## Abstract

In the title compound, [CoCl_2_(C_6_H_8_N_2_)_2_], the Co^II^ ion is four-coordinated by two pyridine N atoms from the 2-amino-3-methyl­pyridine ligands and two chloride ions in a distorted tetra­hedral geometry. A weak intra­molecular N—H⋯Cl inter­action occurs. The crystal packing is stabilized by inter­molecular N—H⋯Cl and C—H⋯Cl hydrogen-bond inter­actions.

## Related literature

2-Amino-3-methyl­pyridine (ampy) can potentially coordinate to metal centers through the N atom of the amino group (Chen *et al.*, 2005 ▶) or the pyridyl nitro­gen atom (Amani Komaei *et al.*, 1999 ▶; Ziegler *et al.*, 2000 ▶; Castillo *et al.*, 2001 ▶). For the structures of [(ampyH)_2_Co*X*_4_] proton-transfer compounds (*X* = Cl, Br), see: Carnevale *et al.* (2010 ▶). Polar metal–halogen bonds are good hydrogen-bond acceptors, see: Aullón *et al.* (1998 ▶).
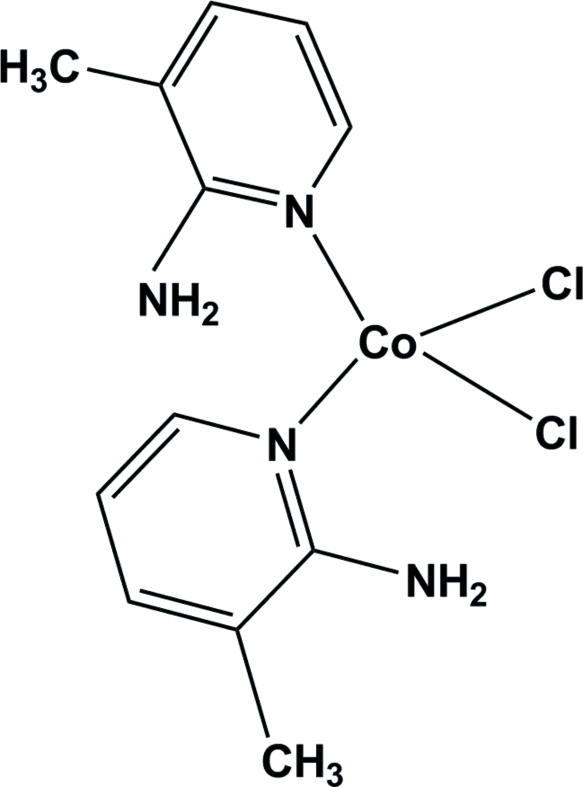


## Experimental

### 

#### Crystal data

[CoCl_2_(C_6_H_8_N_2_)_2_]
*M* *_r_* = 346.12
Monoclinic, 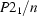

*a* = 9.3768 (19) Å
*b* = 13.841 (3) Å
*c* = 12.175 (2) Å
β = 100.31 (3)°
*V* = 1554.6 (5) Å^3^
*Z* = 4
Mo *K*α radiation
μ = 1.44 mm^−1^
*T* = 298 K
0.50 × 0.38 × 0.30 mm

#### Data collection

Stoe IPDS II diffractometer
Absorption correction: numerical shape of crystal determined optically (*XRED* and *XSHAPE*; Stoe & Cie, 2005 ▶)*T*_min_ = 0.517, *T*_max_ = 0.642
11996 measured reflections
4174 independent reflections
2803 reflections with *I* > 2σ(*I*)
*R*_int_ = 0.055

#### Refinement

*R*[*F* ^2^ > 2σ(*F* ^2^)] = 0.058
*wR*(*F* ^2^) = 0.132
*S* = 1.07
4174 reflections
174 parameters
H-atom parameters constrained
Δρ_max_ = 0.47 e Å^−3^
Δρ_min_ = −0.52 e Å^−3^

### 

Data collection: *X-AREA* (Stoe & Cie, 2005 ▶); cell refinement: *X-AREA*; data reduction: *X-AREA*; program(s) used to solve structure: *SHELXS97* (Sheldrick, 2008 ▶); program(s) used to refine structure: *SHELXL97* (Sheldrick, 2008 ▶); molecular graphics: *ORTEP-3 for Windows* (Farrugia, 1997 ▶); software used to prepare material for publication: *WinGX* (Farrugia, 1999 ▶).

## Supplementary Material

Crystal structure: contains datablocks global, I. DOI: 10.1107/S1600536810036597/jj2058sup1.cif

Structure factors: contains datablocks I. DOI: 10.1107/S1600536810036597/jj2058Isup2.hkl

Additional supplementary materials:  crystallographic information; 3D view; checkCIF report

## Figures and Tables

**Table:** 

Co1—N3	2.034 (2)
Co1—N1	2.038 (3)
Co1—Cl2	2.2303 (11)
Co1—Cl1	2.2635 (11)

**Table:** 

N3—Co1—N1	106.66 (10)
N3—Co1—Cl2	110.23 (8)
N1—Co1—Cl2	111.26 (9)
N3—Co1—Cl1	109.94 (8)
N1—Co1—Cl1	108.24 (8)
Cl2—Co1—Cl1	110.42 (5)

**Table 2:** Hydrogen-bond geometry (Å, °)

*D*—H⋯*A*	*D*—H	H⋯*A*	*D*⋯*A*	*D*—H⋯*A*
N2—H2*B*⋯Cl1^i^	0.86	2.72	3.427 (4)	140
N4—H4*A*⋯Cl1	0.86	2.67	3.363 (4)	138
N4—H4*B*⋯Cl2^ii^	0.86	2.68	3.350 (4)	136
C3—H3⋯Cl2^iii^	0.93	2.81	3.701 (4)	161

Symmetry codes: (i) 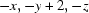; (ii) 
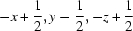
; (iii) 
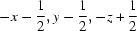
.

**Fig. 1.**
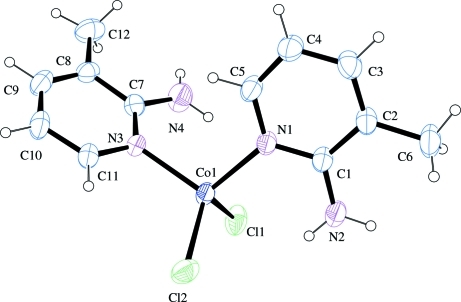
The molecular structure of [Co(ampy)2Cl2] with displacement ellipsoids drawn at 30% probability level.

**Fig. 2.**
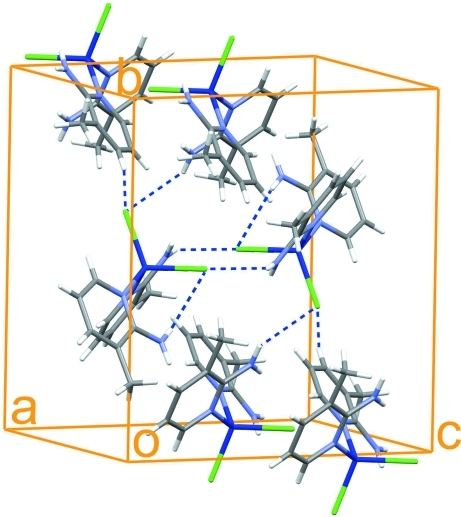
The packing diagram of [Co(ampy)2Cl2] showing hydrogen bonding as blue dashed lines.

## supplementary crystallographic information

### Comment

2-amino-3-methylpyridine (ampy) is a common ligand and potentially can coordinate to metal centers through the N atom of amino group (Chen *et al.*, 2005) or the pyridyl nitrogen atom (Amani Komaei *et al.*, 1999; Ziegler *et al.*, 2000; Castillo *et al.*, 2001). Recently, the structure of [(ampyH)_2_Co*X*_4_] proton transfer compounds (*X*=Cl, Br) have been reported (Carnevale *et al.*, 2010). Polar metal-halogen bonds are good hydrogen bond acceptors (Aullón *et al.*, 1998). We report herein the synthesis and molecular structure of the title compound, [Co(ampy)_2_Cl_2_]. The compound is mononuclear with the cobalt (II) ion coordinated by two pyridyl nitrogen atoms from two ampy ligands and two chloride ions in a distorted tetrahedral geometry (Fig. 1). The Co—N and Co—Cl bond lengths and angles are within normal ranges (Table 1). The dihedral angle formed between the least squares planes of two pyridine rings is 69.5 (5)°. Crystal packing is stabilized by weak intramolecular N–H···Cl and intermolecular N—H···Cl, C—H···Cl hydrogen bond interactions (Table 2). Cl1 forms a bifurcated acceptor bond with H4A and H2B from nearby neighbors (Fig. 2).

### Experimental

A solution of 2-amino-3-methylpyridine (0.1 ml, 1 mmol) in ethanol (10 ml) was added to a solution of CoCl_2_.6H_2_O (0.12 g, 0.5 mmol) in water (10 ml) and stirred for 20 min at 50 °C. Slow evaporation of the resulting solution gave a blue precipitate which was then recrystallized from ethanol and acetonitrile (3:1 *v*/*v*). After one week, blue crystals of the title compound suitable for X-ray analysis were isolated (yield; 0.1583 g, 91.4% based on Co, decomposition > 168 °C).

### Refinement

All of the H atoms were positioned geometrically and refined as riding atoms, with C—H = 0.93Å (CH), with C—H = 0.96Å (CH_3_), and *U*_iso_(H) = 1.2, 1.49*U*_eq_(C), and with N—H = 0.86Å (NH_2_) and *U*_iso_(H) = 1.2*U*_eq_(N).

### Crystal data

**Table:**

[CoCl_2_(C_6_H_8_N_2_)_2_]	*F*(000) = 708.0
*M**_r_* = 346.12	*D*_x_ = 1.479 Mg m^−^^3^
Monoclinic, *P*2_1_/*n*	Melting point: 441 K
Hall symbol: -P 2yn	Mo *K*α radiation, λ = 0.71073 Å
*a* = 9.3768 (19) Å	Cell parameters from 4174 reflections
*b* = 13.841 (3) Å	θ = 2.3–29.2°
*c* = 12.175 (2) Å	µ = 1.44 mm^−^^1^
β = 100.31 (3)°	*T* = 298 K
*V* = 1554.6 (5) Å^3^	Block, blue
*Z* = 4	0.5 × 0.38 × 0.3 mm

### Data collection

**Table:**

Stoe IPDS II diffractometer	4174 independent reflections
Radiation source: fine-focus sealed tube	2803 reflections with *I* > 2σ(*I*)
graphite	*R*_int_ = 0.055
Detector resolution: 0.15 pixels mm^-1^	θ_max_ = 29.2°, θ_min_ = 2.3°
rotation method scans	*h* = −12→10
Absorption correction: numerical shape of crystal determined optically (*X-RED* and *X-SHAPE*; Stoe & Cie, 2005)	*k* = −18→18
*T*_min_ = 0.517, *T*_max_ = 0.642	*l* = −16→16
11996 measured reflections	

### Refinement

**Table:**

Refinement on *F*^2^	Primary atom site location: structure-invariant direct methods
Least-squares matrix: full	Secondary atom site location: difference Fourier map
*R*[*F*^2^ > 2σ(*F*^2^)] = 0.058	Hydrogen site location: inferred from neighbouring sites
*wR*(*F*^2^) = 0.132	H-atom parameters constrained
*S* = 1.07	*w* = 1/[σ^2^(*F*_o_^2^) + (0.0528*P*)^2^ + 0.6654*P*] where *P* = (*F*_o_^2^ + 2*F*_c_^2^)/3
4174 reflections	(Δ/σ)_max_ = 0.001
174 parameters	Δρ_max_ = 0.47 e Å^−^^3^
0 restraints	Δρ_min_ = −0.52 e Å^−^^3^

### Special details

**Table:**

Geometry. All e.s.d.'s (except the e.s.d. in the dihedral angle between two l.s. planes) are estimated using the full covariance matrix. The cell e.s.d.'s are taken into account individually in the estimation of e.s.d.'s in distances, angles and torsion angles; correlations between e.s.d.'s in cell parameters are only used when they are defined by crystal symmetry. An approximate (isotropic) treatment of cell e.s.d.'s is used for estimating e.s.d.'s involving l.s. planes.
Refinement. Refinement of *F*^2^ against ALL reflections. The weighted *R*-factor *wR* and goodness of fit *S* are based on *F*^2^, conventional *R*-factors *R* are based on *F*, with *F* set to zero for negative *F*^2^. The threshold expression of *F*^2^ > σ(*F*^2^) is used only for calculating *R*-factors(gt) *etc*. and is not relevant to the choice of reflections for refinement. *R*-factors based on *F*^2^ are statistically about twice as large as those based on *F*, and *R*- factors based on ALL data will be even larger.

### Fractional atomic coordinates and isotropic or equivalent isotropic displacement parameters (Å^2^)

**Table:**

	*x*	*y*	*z*	*U*_iso_*/*U*_eq_	
Co1	0.14951 (4)	0.99824 (3)	0.31175 (3)	0.04765 (14)	
Cl2	0.09365 (12)	1.14810 (7)	0.35642 (8)	0.0730 (3)	
Cl1	0.24844 (11)	0.99925 (9)	0.15526 (7)	0.0738 (3)	
N3	0.2899 (3)	0.93750 (18)	0.43978 (19)	0.0450 (6)	
N1	−0.0296 (3)	0.9120 (2)	0.2839 (2)	0.0484 (6)	
C5	−0.0369 (4)	0.8380 (3)	0.3553 (3)	0.0579 (8)	
H5	0.0376	0.8310	0.4164	0.069*	
C1	−0.1395 (3)	0.9237 (3)	0.1967 (2)	0.0509 (7)	
C7	0.3589 (3)	0.8548 (2)	0.4302 (3)	0.0490 (7)	
C2	−0.2593 (4)	0.8599 (3)	0.1783 (3)	0.0552 (8)	
C9	0.4738 (4)	0.8617 (3)	0.6192 (3)	0.0641 (9)	
H9	0.5363	0.8367	0.6805	0.077*	
C11	0.3132 (4)	0.9843 (2)	0.5399 (3)	0.0541 (8)	
H11	0.2654	1.0423	0.5464	0.065*	
C6	−0.3807 (4)	0.8754 (4)	0.0808 (3)	0.0829 (13)	
H6A	−0.4567	0.8296	0.0844	0.124*	
H6B	−0.3449	0.8665	0.0124	0.124*	
H6C	−0.4179	0.9398	0.0835	0.124*	
C8	0.4538 (4)	0.8119 (3)	0.5215 (3)	0.0546 (8)	
N2	−0.1321 (4)	0.9997 (2)	0.1281 (3)	0.0756 (10)	
H2A	−0.0593	1.0385	0.1409	0.091*	
H2B	−0.2003	1.0093	0.0718	0.091*	
C10	0.4044 (4)	0.9485 (3)	0.6308 (3)	0.0664 (10)	
H10	0.4195	0.9814	0.6985	0.080*	
C3	−0.2594 (4)	0.7855 (3)	0.2517 (3)	0.0680 (10)	
H3	−0.3359	0.7418	0.2410	0.082*	
C4	−0.1470 (4)	0.7738 (3)	0.3422 (3)	0.0705 (10)	
H4	−0.1477	0.7232	0.3923	0.085*	
N4	0.3322 (4)	0.8108 (3)	0.3292 (3)	0.0793 (10)	
H4A	0.2730	0.8365	0.2749	0.095*	
H4B	0.3745	0.7572	0.3193	0.095*	
C12	0.5285 (5)	0.7188 (3)	0.5056 (4)	0.0809 (12)	
H12A	0.5894	0.7005	0.5744	0.121*	
H12B	0.5866	0.7267	0.4488	0.121*	
H12C	0.4574	0.6693	0.4833	0.121*	

### Atomic displacement parameters (Å^2^)

**Table:**

	*U*^11^	*U*^22^	*U*^33^	*U*^12^	*U*^13^	*U*^23^
Co1	0.0456 (2)	0.0545 (2)	0.0391 (2)	−0.0008 (2)	−0.00267 (15)	0.00612 (18)
Cl2	0.0862 (7)	0.0581 (5)	0.0698 (5)	0.0198 (5)	0.0008 (5)	0.0085 (4)
Cl1	0.0666 (5)	0.1115 (8)	0.0421 (4)	−0.0138 (6)	0.0066 (4)	0.0074 (5)
N3	0.0440 (13)	0.0488 (14)	0.0392 (12)	0.0019 (11)	−0.0004 (10)	0.0034 (10)
N1	0.0436 (14)	0.0585 (15)	0.0405 (12)	−0.0034 (11)	0.0005 (10)	0.0044 (11)
C5	0.0514 (18)	0.075 (2)	0.0442 (16)	−0.0008 (17)	0.0012 (14)	0.0128 (15)
C1	0.0452 (16)	0.0637 (19)	0.0419 (15)	−0.0024 (14)	0.0024 (13)	0.0021 (14)
C7	0.0448 (16)	0.0516 (17)	0.0499 (16)	−0.0021 (14)	0.0059 (13)	−0.0013 (14)
C2	0.0454 (16)	0.076 (2)	0.0431 (16)	−0.0095 (16)	0.0038 (13)	0.0002 (15)
C9	0.058 (2)	0.078 (2)	0.0525 (19)	0.0033 (18)	−0.0009 (16)	0.0191 (18)
C11	0.0531 (17)	0.060 (2)	0.0454 (15)	−0.0001 (15)	−0.0011 (13)	−0.0006 (14)
C6	0.057 (2)	0.121 (4)	0.062 (2)	−0.025 (2)	−0.0139 (18)	0.015 (2)
C8	0.0425 (15)	0.0540 (18)	0.066 (2)	−0.0001 (14)	0.0062 (14)	0.0155 (16)
N2	0.0690 (19)	0.082 (2)	0.0641 (18)	−0.0192 (17)	−0.0205 (15)	0.0253 (17)
C10	0.070 (2)	0.084 (3)	0.0393 (16)	−0.002 (2)	−0.0054 (15)	−0.0015 (17)
C3	0.056 (2)	0.086 (3)	0.061 (2)	−0.023 (2)	0.0079 (16)	0.0053 (19)
C4	0.059 (2)	0.085 (3)	0.065 (2)	−0.015 (2)	0.0053 (17)	0.024 (2)
N4	0.093 (2)	0.075 (2)	0.0648 (19)	0.0253 (19)	−0.0001 (17)	−0.0170 (16)
C12	0.074 (3)	0.067 (2)	0.102 (3)	0.019 (2)	0.016 (2)	0.019 (2)

### Geometric parameters (Å, °)

**Table:**

Co1—N3	2.034 (2)	C9—H9	0.9300
Co1—N1	2.038 (3)	C11—C10	1.365 (5)
Co1—Cl2	2.2303 (11)	C11—H11	0.9300
Co1—Cl1	2.2635 (11)	C6—H6A	0.9600
N3—C7	1.330 (4)	C6—H6B	0.9600
N3—C11	1.363 (4)	C6—H6C	0.9600
N1—C1	1.350 (4)	C8—C12	1.495 (6)
N1—C5	1.353 (4)	N2—H2A	0.8600
C5—C4	1.349 (5)	N2—H2B	0.8600
C5—H5	0.9300	C10—H10	0.9300
C1—N2	1.352 (4)	C3—C4	1.391 (5)
C1—C2	1.415 (5)	C3—H3	0.9300
C7—N4	1.355 (4)	C4—H4	0.9300
C7—C8	1.424 (4)	N4—H4A	0.8600
C2—C3	1.363 (5)	N4—H4B	0.8600
C2—C6	1.506 (5)	C12—H12A	0.9600
C9—C8	1.358 (5)	C12—H12B	0.9600
C9—C10	1.385 (6)	C12—H12C	0.9600
			
N3—Co1—N1	106.66 (10)	C2—C6—H6A	109.5
N3—Co1—Cl2	110.23 (8)	C2—C6—H6B	109.5
N1—Co1—Cl2	111.26 (9)	H6A—C6—H6B	109.5
N3—Co1—Cl1	109.94 (8)	C2—C6—H6C	109.5
N1—Co1—Cl1	108.24 (8)	H6A—C6—H6C	109.5
Cl2—Co1—Cl1	110.42 (5)	H6B—C6—H6C	109.5
C7—N3—C11	119.0 (3)	C9—C8—C7	116.1 (3)
C7—N3—Co1	123.1 (2)	C9—C8—C12	123.8 (3)
C11—N3—Co1	117.8 (2)	C7—C8—C12	120.0 (3)
C1—N1—C5	118.5 (3)	C1—N2—H2A	120.0
C1—N1—Co1	123.4 (2)	C1—N2—H2B	120.0
C5—N1—Co1	118.1 (2)	H2A—N2—H2B	120.0
C4—C5—N1	123.2 (3)	C11—C10—C9	118.0 (3)
C4—C5—H5	118.4	C11—C10—H10	121.0
N1—C5—H5	118.4	C9—C10—H10	121.0
N1—C1—N2	117.6 (3)	C2—C3—C4	121.1 (3)
N1—C1—C2	121.4 (3)	C2—C3—H3	119.4
N2—C1—C2	121.0 (3)	C4—C3—H3	119.4
N3—C7—N4	117.0 (3)	C5—C4—C3	118.2 (3)
N3—C7—C8	122.4 (3)	C5—C4—H4	120.9
N4—C7—C8	120.6 (3)	C3—C4—H4	120.9
C3—C2—C1	117.5 (3)	C7—N4—H4A	120.0
C3—C2—C6	122.3 (3)	C7—N4—H4B	120.0
C1—C2—C6	120.2 (3)	H4A—N4—H4B	120.0
C8—C9—C10	122.4 (3)	C8—C12—H12A	109.5
C8—C9—H9	118.8	C8—C12—H12B	109.5
C10—C9—H9	118.8	H12A—C12—H12B	109.5
N3—C11—C10	122.0 (3)	C8—C12—H12C	109.5
N3—C11—H11	119.0	H12A—C12—H12C	109.5
C10—C11—H11	119.0	H12B—C12—H12C	109.5
			
N1—Co1—N3—C7	69.9 (3)	C11—N3—C7—C8	1.6 (5)
Cl2—Co1—N3—C7	−169.2 (2)	Co1—N3—C7—C8	−178.2 (2)
Cl1—Co1—N3—C7	−47.3 (3)	N1—C1—C2—C3	−0.4 (5)
N1—Co1—N3—C11	−109.9 (2)	N2—C1—C2—C3	−179.4 (4)
Cl2—Co1—N3—C11	11.0 (3)	N1—C1—C2—C6	179.5 (4)
Cl1—Co1—N3—C11	133.0 (2)	N2—C1—C2—C6	0.5 (6)
N3—Co1—N1—C1	−172.4 (3)	C7—N3—C11—C10	−0.4 (5)
Cl2—Co1—N1—C1	67.4 (3)	Co1—N3—C11—C10	179.3 (3)
Cl1—Co1—N1—C1	−54.1 (3)	C10—C9—C8—C7	1.1 (5)
N3—Co1—N1—C5	6.1 (3)	C10—C9—C8—C12	179.1 (4)
Cl2—Co1—N1—C5	−114.1 (2)	N3—C7—C8—C9	−1.9 (5)
Cl1—Co1—N1—C5	124.4 (2)	N4—C7—C8—C9	179.8 (4)
C1—N1—C5—C4	1.6 (5)	N3—C7—C8—C12	−180.0 (3)
Co1—N1—C5—C4	−176.9 (3)	N4—C7—C8—C12	1.8 (5)
C5—N1—C1—N2	178.2 (3)	N3—C11—C10—C9	−0.3 (6)
Co1—N1—C1—N2	−3.3 (4)	C8—C9—C10—C11	−0.1 (6)
C5—N1—C1—C2	−0.9 (5)	C1—C2—C3—C4	1.1 (6)
Co1—N1—C1—C2	177.6 (2)	C6—C2—C3—C4	−178.9 (4)
C11—N3—C7—N4	179.9 (3)	N1—C5—C4—C3	−1.0 (6)
Co1—N3—C7—N4	0.1 (4)	C2—C3—C4—C5	−0.4 (7)

### Hydrogen-bond geometry (Å, °)

**Table:**

*D*—H···*A*	*D*—H	H···*A*	*D*···*A*	*D*—H···*A*
N2—H2B···Cl1^i^	0.86	2.72	3.427 (4)	140
N4—H4A···Cl1	0.86	2.67	3.363 (4)	138
N4—H4B···Cl2^ii^	0.86	2.68	3.350 (4)	136
C3—H3···Cl2^iii^	0.93	2.81	3.701 (4)	161

Symmetry codes: (i) −*x*, −*y*+2, −*z*; (ii) −*x*+1/2, *y*−1/2, −*z*+1/2; (iii) −*x*−1/2, *y*−1/2, −*z*+1/2.

## References

[bb1] Amani Komaei, S., Van Albada, G. A., Mutikainen, I., Turpeinen, U. & Reedijk, J. (1999). *Polyhedron*, **18**, 1991–1997.

[bb2] Aullón, G., Bellamy, D., Brammer, L., Bruton, E. A. & Orpen, A. G. (1998). *Chem. Commun.* pp. 653–654.

[bb3] Carnevale, D. J., Landee, C. P., Turnbull, M. M., Winn, M. & Xiao, F. (2010). *J. Coord. Chem.* **63**, 2223–2238.

[bb4] Castillo, O., Luque, A., Lloret, F. & Román, P. (2001). *Inorg. Chem. Commun.* **4**, 350–353.

[bb5] Chen, Z.-F., Liu, B., Liang, H., Hu, R.-X. & Zhou, Z.-Y. (2005). *J. Coord. Chem.* **28**, 561–565.

[bb6] Farrugia, L. J. (1997). *J. Appl. Cryst.* **30**, 565.

[bb7] Farrugia, L. J. (1999). *J. Appl. Cryst.* **32**, 837–838.

[bb8] Sheldrick, G. M. (2008). *Acta Cryst.* A**64**, 112–122. 10.1107/S0108767307043930 18156677

[bb9] Stoe & Cie (2005). *X-AREA*, *X-RED* and *X-SHAPE* Stoe & Cie, Darmstadt, Germany.

[bb10] Ziegler, C. J., Silverman, A. P. & Lippard, S. J. (2000). *J. Biol. Inorg. Chem.* **5**, 774–783. 10.1007/s007750000170 11129005

